# Low-kVp techniques for radiation dose optimization in abdominopelvic contrast-enhanced CT: a scoping review

**DOI:** 10.1007/s12194-026-01063-9

**Published:** 2026-06-03

**Authors:** Nitika C. Panakkal, Rajagopal Kadavigere, Suresh Sukumar, N. Ravishankar, Shivanath Shanbhag, Visakh Thrivikraman Nair

**Affiliations:** 1 Department of Medical Imaging Technology, Manipal College of Health Professions, Manipal Academy of Higher Education, Manipal, Karnataka India; 2 Department of Radio diagnosis & Medical Imaging, Kasturba Medical College, Manipal Academy of Higher Education, Manipal, Karnataka India; 3 Department of Biostatistics, Vallabhbhai Patel Chest Institute, University of Delhi, New Delhi, India

**Keywords:** Low-kVp CT, radiation dose optimization, abdominopelvic CT

## Abstract

Contrast-enhanced computed tomography (CECT) of the abdomen and pelvis is widely used for diagnostic imaging but contributes substantially to cumulative medical radiation exposure. Low tube voltage (kVp) imaging has gained attention as a practical strategy for radiation dose optimization while maintaining diagnostic image quality.The study aimed to map the current evidence on low-kVp techniques for radiation dose reduction in adult abdominopelvic CECT and evaluate their effects on radiation dose and image quality. This scoping review was conducted in accordance with the Arksey and O’Malley framework and PRISMA-ScR guidelines. A systematic search of Scopus, PubMed, Web of Science, Cochrane Library, and CINAHL identified studies published between 2000 and 2025 that reported low-kVp dose optimization strategies in adult patients. Data were extracted on scanner characteristics, tube voltage settings, reconstruction techniques, and reported dose reductions. Thirteen studies (sample size: 30–907) patients were included. Tube voltage settings ranged from 80 to 120 kVp, with 80 kVp consistently achieving substantial dose reductions (11–86%), particularly when combined with hybrid, model-based, or deep learning reconstruction algorithms that mitigated image noise and preserved diagnostic confidence. Automatic tube voltage selection and patient size–adapted protocols further supported individualized dose optimization. However, considerable heterogeneity in imaging protocols, reconstruction methods, and dose metrics limited comparability across studies. Low-kVp imaging is an effective approach for reducing radiation dose in contrast-enhanced abdominopelvic CT without compromising diagnostic adequacy. Standardized reporting and large prospective multicenter studies are needed to establish optimal protocols and support broader clinical implementation.

## Introduction

Computed tomography (CT) is considered one of the most significant contributors to medical radiation exposure and is widely used for diagnosing abdominal and pelvic conditions due to its high spatial resolution and diagnostic accuracy. However, this widespread use is accompanied by significant concerns regarding the associated radiation dose, which is especially important given the radiosensitivity of many organs in the abdomen and pelvis [1]. According to published data, around 70% of the radiation dose is obtained from CT scans for patients undergoing medical imaging procedures [2, 3]. Furthermore, 47% of the radiation doses are from CT scans of the abdomen and pelvis [4, 5]. CT scans of the abdomen and pelvis have been consistently associated with higher radiation doses relative to other anatomical regions, mainly due to larger scan coverage and multiphasic acquisitions [6, 7], Moreover, many adult patients undergo multiple imaging studies for chronic conditions such as cancer, gastrointestinal diseases, or follow-up of surgical procedures, making the cumulative dose a growing concern. Therefore, it is essential to adopt dose reduction strategies that maintain diagnostic accuracy while minimizing radiation exposure, in line with as low as reasonably achievable (ALARA) [8, 9]. Various optimization techniques have been applied to reduce radiation dose in CT examinations of the abdomen and pelvis. These include adjusting exposure parameters such as kVp, mAs, using tube current modulation, automatic tube voltage selection as per body size, scanning parameters like scan range, pitch, gantry rotation time, and using dose monitoring software [10, 11]. Among these strategies, reducing tube voltage remains one of the most established and effective approaches [12]. This is because radiation output is directly related to tube voltage. Lowering the kVp enhances the photoelectric effect, which is more dominant at lower photon energies. In the presence of iodinated contrast media, this leads to increased iodine attenuation and consequently improved tissue and lesion contrast in contrast-enhanced CT (CECT) images [13–15]. CT radiation dose is strongly influenced by patient body mass index (BMI), particularly in individuals with a low BMI, where identical scan settings can result in disproportionately high radiation exposure, thereby increasing potential health risks [16]. Additionally, BMI is a key factor in determining the appropriate selection of tube voltage. However, when other scanning parameters are held constant, reducing tube voltage decreases X-ray energy and penetration, which can lead to increased image noise and potentially lower image quality, thus affecting diagnostic accuracy [17].

Early multidetector CT systems were constrained by limited tube current output and reliance on filtered back projection (FBP), which restricted the clinical feasibility of low-kVp protocols because of excessive image noise [18, 19]. Subsequent advances in X-ray tube design enable substantially higher tube current capabilities, allowing adequate photon flux to be achieved even at lower tube voltages [20, 21]. The introduction of automatic exposure control and patient size-adapted acquisition strategies further improved dose efficiency and supported individualized tube voltage selection [22, 23]. To counter increased image noise from low tube voltage, adaptive statistical iterative reconstruction (ASIR-V), an advanced iterative reconstruction algorithm, uses improved noise, object, and physics models to balance noise reduction and spatial resolution better [24]. More broadly, the transition from FBP to hybrid and model-based iterative reconstruction (MBIR) techniques substantially reduced image noise and artifacts, making low-kVp imaging clinically viable in routine practice [25, 26]. More recently, deep learning-based reconstruction (DLR) methods have further enhanced noise suppression and spatial resolution, enabling greater reductions in radiation dose and contrast medium volume while preserving diagnostic confidence [27].

Although low-kVp CT techniques have been discussed in prior reviews and incorporated into clinical guidelines, the literature specific to CECT abdominopelvic remains heterogeneous. While major guidelines acknowledge tube voltage adaptation and contrast dose optimization [28], CT protocols vary widely across clinical practices, reflecting differences in scanner technology, acquisition parameters, reconstruction methods, radiation dose descriptors, and image quality assessment approaches, thereby complicating standardization and routine implementation in clinical workflows [29]. Moreover, much of the evidence supporting low-kVp imaging is derived from angiographic or vascular-focused applications, where high iodine contrast and relatively large target structures predominate, whereas fewer studies have systematically evaluated low-kVp protocols in routine abdominopelvic CT, particularly for the detection and characterization of small or low-contrast lesions [30]. Given the high radiation burden, multiphasic nature, and unique diagnostic requirements of abdominopelvic CT, evidence from other anatomical regions cannot be directly extrapolated. A scoping review is therefore appropriate to systematically map how low-kVp techniques have been applied in adult abdominopelvic CECT and to identify gaps in the existing evidence.

The objective is to map and synthesize the existing literature on the use of low tube voltage techniques for radiation dose optimization in CECT imaging of the abdomen and pelvis in adult patients, with a focus on their impact on radiation dose and diagnostic image quality.

### Methods and framework

This scoping review was conducted following the Arksey and O’Malley framework [31] and in accordance with the PRISMA–ScR guidelines [32]. This review followed five Key stages:1) Formulation of the research question,2) identification of relevant studies,3) selection of studies based on predefined criteria, 4) data charting and extraction,5) synthesis and summarization, and reporting of the findings.

### Identifying the research question

What radiation dose optimization strategies involving low tube voltage have been reported in the literature for CECT of the abdomen and pelvis in adult patients, and how do these strategies impact radiation dose and diagnostic image quality?

#### Literature search strategy

## Search strategies

A comprehensive search was performed across five peer-reviewed databases: Scopus, PubMed, Web of Science, Cochrane Library, and CINAHL. The search covered literature published between 2000 and 2025. Table 1 presents the MeSH terms used, along with appropriate Boolean operators (“And”, “OR”) and wildcards where applicable.

### Study eligibility

Studies were screened specifically for those addressing radiation dose optimization in CECT of the abdomen and pelvis. The inclusion criteria comprised articles that provided information on low-kVp radiation dose reduction techniques. Articles were excluded if they were not in English, not available in full text, not open access, or if they were conference papers or under review.

## Study selection and data extraction

**Fig. 1:**
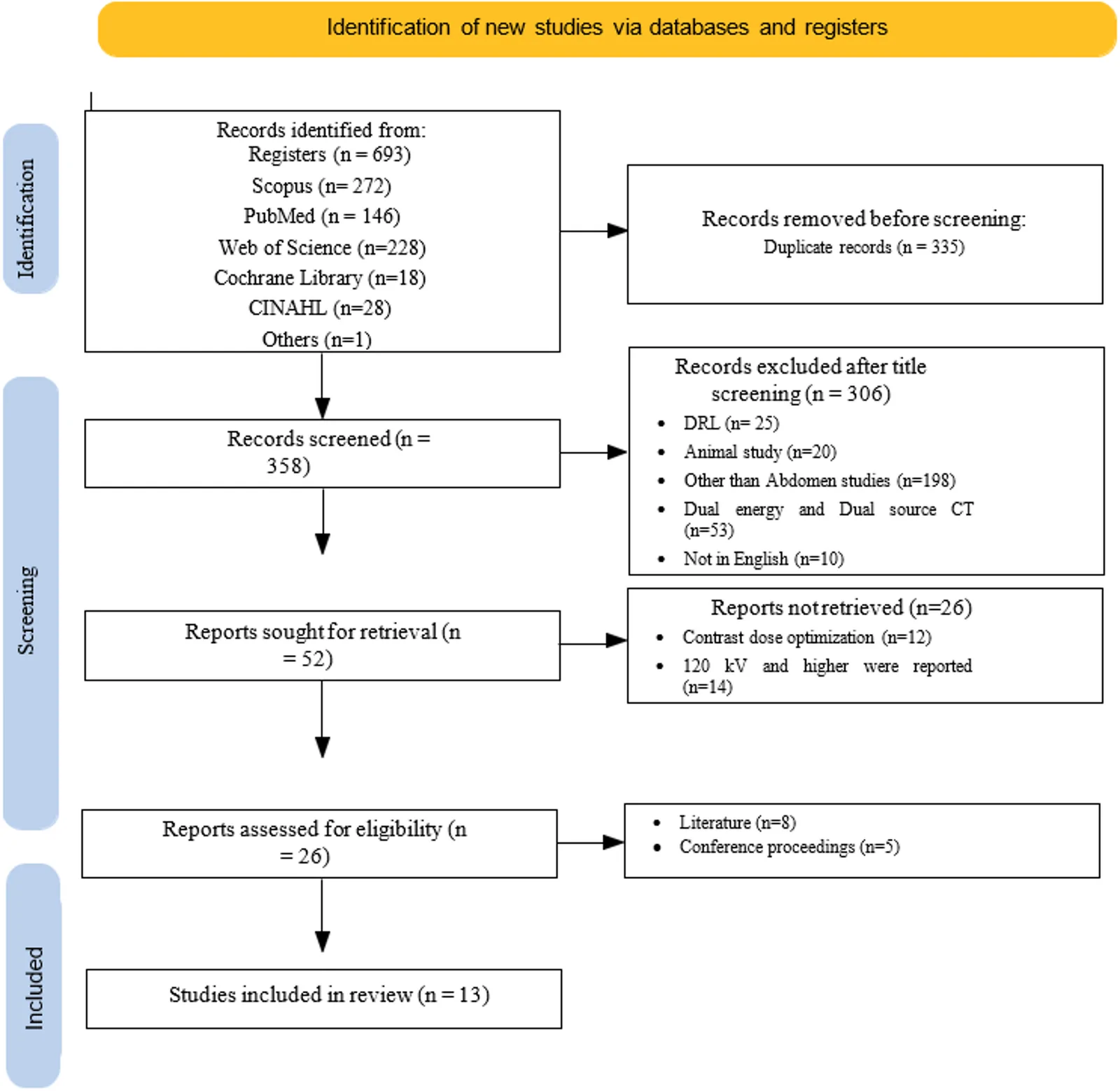
PRISMA–ScR flow diagram

The search results were imported into the Rayyan software for screening and de-duplication. Titles and abstracts of all retrieved studies were independently and blindly screened by two reviewers (NP and SSH). Any discrepancies were resolved through discussion, and when necessary, a third reviewer (RKV) was consulted to reach consensus. Studies were excluded if the titles indicated that they focused on diagnostic reference levels, anatomical regions other than the abdomen, or involved dual-energy or dual-source CT (Fig. 1). The full texts of potentially eligible articles were then reviewed in detail, and relevant data were extracted using a structured data charting table.

**Table 1.** PICO framework with corresponding MeSH terms used in database search

Population	Intervention	Comparison	Outcome
Computed Tomography Abdomen OR abdomen	Low-kVp Low Tube voltage Radiation dose optimization	Not applicable	Radiation Dosage Image Quality Diagnostic Accuracy

## Data charting process

A structured data charting approach was employed to extract key variables from each included study. As technical parameters and reconstruction methods directly influence radiation dose and image quality, the following variables were collected: sample size to facilitate comparison across heterogeneous evidence, CT vendor, and model to account for inter-scanner variability in acquisition and reconstruction technique due to its impact on image quality and noise characteristics. Tube voltage (kVp), and tube current (mAs) were documented as primary determinants of radiation dose. Clinical indication and imaging phase were included to capture protocol variability across different diagnostic contexts. Radiation dose metrics, including volume computed tomography dose index (CTDI_vol_), dose length product (DLP), effective dose, or size-specific dose estimate (SSDE), were extracted to enable standardized comparison of radiation exposure. Additionally, percentage dose reduction was recorded to evaluate the effectiveness of dose optimization strategies. The extracted data are summarized in Table 2.

**Table 2:** Key study parameters and dose reduction outcomes in low-kVp CT imaging

Author(s) (Year)	Sample size	CT Vendor & model	Reconstruction technique	kVp	mAs or reference mAs	Indication	Phase	Radiation dose reduction %
Yekeler et al.[33] 2005	76	Siemens SOMATOM Sensation 4-row	FBP	80, 120	95 (80 kV), 30 (120 kV)	Urinary stone	Non-contrast phase	NR
Yanaga et al.[34] 2011	111	Philips Brilliance 64-MDCT	Standard FBP	80, 120	600 mAs (80 kVp),300 mAs + AEC (120 kVp)	HCC	UE, AP, PP, equilibrium phases	12.9% ED
Cho et al.[35] 2012	30	Siemens Somatom Sensation 16	FBP	80, 100, 120, 140	150	Abdominal cancer	AP, VP, DP	36% (CTDI_vol_)
Ichikawa et al.[36] 2013	128	Toshiba Aquilion ONE (320-row for 80 kVp), 64-slice for 120 kVp	Filtered Back Projection	80, 120	280–450 (auto)	HCC	Hepatic arterial phase	84.5% (CTDI_vol_), 86.0% (DLP)
Hardie et al.[37] 2013	41	Siemens SOMATOM Definition Flash	FBP, SAFIRE	100, 120	SAFIRE: 150, FBP: 190–320	Routine contrast-enhanced abdominopelvic CT	PP	Up to 50% (CTDI_vol_)
Park et al.[38] 2017	97	Toshiba Aquilion ONE (320-row)	Adaptive Iterative Dose Reduction 3D	80	244–337	Liver dynamic CT	Arterial phase	NR
Knipp et al.[39] 2017	103 (49 at 80 kV, 54 at 120 kV)	Philips Brilliance iCT or 64	iDose (Hybrid IR), FBP	80, 120	504 (80 kV), 243 (120 kV)	Azotemic Patients	Arterial phase	43% (ED)
Motlagh et al.[40] 2018	40	Toshiba Activion 16	FBP	80, 120	50 (80 kV), auto (120 kV)	Detection of illicit drug packets	Non-contrast phase	9.7 times lower dose compared to the standard protocols
Moloney et al.[41] 2018	57	GE Discovery CT 750 HD	CD: 40% ASIR, LD: MBIR	100	LD: 20–350; CD: 50–350 (ATCM)	Acute abdominal pain	Single contrast-enhanced phase (45 s)	74.7% mean ED reduction
Choi et al.[42] 2020	907	Siemens SOMATOM Definition AS+ (128-channel)	Filtered Back Projection	80, 100, 120	578 (80 kV), 248 (100 kV), 180 (120 kV)	Gastric Cancer Surveillance	Portal venous phase CT	15.4% (CTDI_vol_), 11% (DLP)
Yoshida et al.[43] 2024	148	Canon, Aquilion One (320-row)	120-kVp: Hybrid IR (AIDR 3D); 80-kVp: HIR & Deep-Learning Recon (AiCE)	80, 120	Auto (284–800); 120–500	Oncologic evaluation, acute abdomen, trauma	Single contrast-enhanced phase (80 s)	40.4% (SSDE),
Zhu et al.[44] 2024	60	United Imaging Healthcare uCT 960+	HIR; Deep Learning Iterative Recon (Deep IR, Levels 1–5)	80, 100	Ref: 213 (100 kV), 130 (80 kV)	upper abdominal enhanced CT examination	AP, PP, DP	69.94% (ED)
Panakkal et al. [45] 2025	216 (120 SP, 96 LDP)	Philips Incisive CT (128-slice)	Hybrid iterative reconstruction (iDose4, level 3)	80, 100,120	ATCM: 80–350 mA (low BMI), 80–250 mA (normal BMI), 80–350 mA (high BMI	Routine contrast-enhanced abdominopelvic CT	UE, AP, PP, and 3 min delay	70.9% (low BMI), 44.4% (normal BMI), 38.0% (high BMI) (ED)

CTDI_vol_* (mGy) volume computed tomography dose index represents scanner output, DLP* (mGy.cm) dose-length product reflects the total examination dose, ED* (mSv) effective dose is a population-based radiation risk estimate derived from DLP, SSDE* (mGy) size-specific dose estimate adjusts CTDI_vol_ for patient size, HCC*  hepatocellular carcinoma, UE*  un-enhanced phase, AP* arterial phase, PP* portal venous phase, VP* venous phase, DP* delayed phase

## Results

The results are presented according to the predefined data items extracted during the data charting process, including study characteristics, CT system parameters, acquisition parameters, and reconstruction techniques.

## Study characteristics

A total of 13 studies were included in this scoping review, encompassing a range of study designs and sample sizes, with populations ranging from 30 to 907 patients. These studies were published between 2005 and 2025, and the research was conducted across multiple countries, reflecting a global interest in low-kVp CT dose optimization strategies.

## CT system characteristics

Multiple CT scanner vendors were reported, including Canon, Siemens, Philips, GE, and United Imaging Healthcare. The scanner types varied from 16-slice to 320-row multidetector CT systems, demonstrating that low-kVp protocols are being explored across a wide range of scanner capabilities.

### Acquisition parameters

The studies included utilized different tube voltage settings that varied from 80 to 140 kVp [15, 35, 46], with most comparisons focusing on 80, 100, and 120 kVp. Among these, 80 kVp was most frequently used to achieve dose reduction [15, 33–35, 38–40, 42–44], while 100 kVp served as an intermediate option, balancing dose and image quality [15, 35, 37, 41, 42, 44, 46]. 120 kVp was commonly used as the standard reference protocol [15, 33–37, 39, 40, 42, 43, 46]. Tube current values, including both automatic exposure control and fixed reference settings, varied considerably across studies. Automatic modulation systems typically operated within ranges such as 284–800 mAs [43] or 280–450 mAs [36]. For fixed protocols, low-dose techniques generally use settings between 50 [40] and 150 mAs [35], whereas standard-dose protocols went up to 800 mAs [43].

### Reconstruction techniques

A diverse range of reconstruction algorithms was employed across the included studies to offset the increased image noise associated with low-kVp imaging. FBP was used as a baseline technique, particularly in conventionally designed protocols [15, 33–37, 39, 40, 42]. Hybrid iterative reconstruction (HIR), including algorithms such as adaptive iterative dose reduction three-dimensional (AIDR 3D), iDose, and Sinogram Affirmed Iterative reconstruction (SAFIRE), was widely adopted to enhance image quality while reducing noise [37,39, 43, 44, 46]. MBIR enabled substantial radiation dose reductions while preserving diagnostic performance. More recently, DLR-based techniques such as Advanced intelligent Clear-IQ Engine (AiCE) and deep IR have been increasingly utilized, demonstrating notable improvements in image quality and dose efficiency [41, 43, 44].

### Radiation dose outcomes and image quality

The included studies reported substantial reductions in radiation dose with low-kVp protocols. Dose reductions ranged from approximately 11% to over 86%, depending on the protocol, scanner technology, and reconstruction method. A recent BMI-adapted protocol demonstrated dose reductions ranging from 38 to 70.9% across different BMI categories while maintaining acceptable diagnostic image quality [45]. Studies using 80 kVp commonly demonstrated higher dose reduction compared to standard 120 kVp protocols. Image quality was evaluated using quantitative metrics such as image noise, signal-to-noise ratio (SNR), and contrast-to-noise ratio (CNR). While lower kVp increased image noise, the use of iterative and deep learning–based reconstruction techniques resulted in measurable improvements in SNR and CNR, thereby maintaining diagnostic image quality.

### Clinical indication and imaging phases

The included studies covered a range of clinical indications, including hepatocellular carcinoma evaluation, abdominal malignancies, acute abdominal pain, oncologic assessment, and urinary stone detection. Most studies employed CECT protocols with multiphasic acquisitions, including arterial, portal venous, and delayed phases, while some utilized single-phase imaging depending on the clinical indication.

## Discussion

The tube voltage is a key parameter influencing both radiation dose and image quality. The dose increases approximately with the square of the kVp, making small reductions in tube voltage highly effective for dose reduction [47]. Lowering kVp also enhances soft tissue and lesion contrast in CECT due to increased attenuation of contrast medium [47, 48], through it can increase image noise, particularly in larger patients [49, 50].

### Dose reduction and image quality preservation with low-kVp CT

Studies consistently demonstrate that reducing tube voltage results in a substantial reduction in radiation dose while maintaining diagnostic image quality [35, 51]. Although lower kVp inherently increases image noise, advanced reconstruction techniques, including iterative and deep learning-based methods, effectively mitigate this effect and preserve diagnostic quality [52-55]. Across studies, image quality was assessed using both quantitative metrics, such as image noise, SNR, and CNR, and qualitative radiologist evaluation to confirm diagnostic adequacy [54, 56]. These findings suggest that low-kVp imaging can achieve an appropriate balance between radiation dose reduction and diagnostic performance when combined with suitable reconstruction strategies [40, 42]. Automatic tube voltage selection generally provides more modest dose savings while maintaining or improving CNR [42]. More advanced strategies, including volumetric dynamic CT at low tube voltage and deep learning–based iterative reconstruction, have demonstrated substantial improvements in dose efficiency while preserving diagnostic image quality [36, 44]. Similarly, patient size–adjusted dose assessment using SSDE has shown clear benefits in optimizing radiation dose while maintaining image quality in low-kVp protocols [43].

### Manual and automatic tube voltage selection

Manual adjustment of tube voltage, particularly when tailored to patient size or BMI, has been shown to reduce radiation exposure while maintaining acceptable image quality [39, 57]. Ultra-low-dose protocols using reduced tube voltage have also demonstrated feasibility while preserving diagnostic acceptability [40]. BMI-adapted protocols further support the role of patient-specific optimization and may also enable a reduction in contrast media volume [58]. BMI-specific tube voltage selection, combined with ATMC, highlights the feasibility of individualized protocol optimization, enabling dose reduction through coordinated adjustment of acquisition parameters [45]. Automatic tube voltage selection, typically operating within a range of 80–120 kVp, enables standardized, patient-specific adjustment based on attenuation and CNR characteristics [59]. However, evidence from large-scale analyses suggests that tube voltage modulation alone may contribute less to overall dose variability in routine CT practice, where factors such as tube current and the number of acquisition phases play a more significant role [60].

### Role of patient size, BMI, and body habitus

Body mass index has been widely used as a reference standard for stratifying patients in dose reduction protocols for abdominal CT. Several studies have shown that BMI plays an important role in determining image quality metrics such as image noise and CNR [61-63]. While patient size is commonly assessed using BMI, alternative body habitus parameters may also influence image quality. For example, muscle volume has been reported to have a greater impact on image noise during low-kVp CT compared with abdominal fat, particularly in smaller individuals [38]. These findings suggest that body composition parameters, such as muscle volume, may provide additional value beyond BMI in patient-specific dose optimization. Incorporating body habitus into techniques such as automatic exposure control (AEC) or automatic tube voltage selection (ATVS) could improve personalization of CT protocols. To mitigate increased noise in low-kVp imaging, higher tube currents have been employed in some protocols [54, 64]. These findings highlight the importance of adjusting acquisition parameters based on patient size, particularly when employing low-kVp techniques, which can be further optimized through advanced reconstruction methods. BMI-adapted protocols demonstrate that tailoring tube voltage according to patient size can achieve significant radiation dose reductions across different patient groups, although increased image noise may remain a consideration in higher BMI individuals [45]. The combination of low tube voltage with iterative and deep learning-based reconstruction has demonstrated improved dose efficiency while maintaining diagnostic quality, especially in smaller patients [41, 44]. Additionally, low-kVp protocols are feasible in appropriately selected patient populations without compromising diagnostic adequacy, although increased image noise remains a consideration [36, 55]. These results emphasize the need for patient-specific optimization when implementing low-kVp CT protocols.

### Image quality assessment and diagnostic adequacy

Across the included studies, the diagnostic adequacy of low-kVp CT was evaluated using both quantitative and qualitative image quality criteria. Quantitative assessment commonly included measurements of image noise, SNR, and CNR in solid organs and vascular structures, whereas qualitative assessment relied on radiologist evaluation of image visualization, critical reproduction of anatomical structures, image noise, and artifacts using 3-to-5-point Likert scales or guideline-based criteria [56]. Despite increased image noise at lower tube voltages, most studies reported preserved or improved diagnostic confidence when iterative or deep learning-based reconstruction techniques were applied, indicating that dose reduction was achieved without compromising clinical interpretability [15, 36, 42].

However, emerging task-based evidence suggests that improvements in perceptual image quality do not always translate into preserved diagnostic performance. Jensen et al. demonstrated that at substantially reduced radiation doses, deep learning-based reconstruction was associated with significantly lower detectability and inferior characterization of small low-contrast liver lesions (< 0.5 cm) compared with standard-dose CT reconstructed using filtered back projection. Importantly, this reduction in lesion detectability occurred despite higher subjective image quality scores and improved contrast-to-noise ratios. Although their study did not directly compare tube voltages, it provides clinically relevant evidence that aggressive dose reduction has definable diagnostic limits, even when advanced reconstruction algorithms are applied. These findings highlight a critical limitation of relying solely on conventional image quality metrics. Improvements in noise reduction or visual image quality do not necessarily correspond to improved lesion detectability or diagnostic performance. Therefore, task-based evaluation, which assesses the ability to detect and characterize clinically relevant lesions, is essential when evaluating low-kVp CT protocols. Such approaches provide a more clinically meaningful assessment compared with traditional image quality metrics alone [65].

### Dual-energy CT in Relation to low-kVp imaging

Although dual energy CT (DECT) was excluded from the scope of the review, approximately 15% of the identified records demonstrated dose reduction with dual energy. DECT is closely related to low-kVp imaging from both physical and technical perspectives. Many DECT implementations incorporate low-kVp acquisitions to exploit increased iodine attenuation at lower photon energies, thereby enhancing contrast conspicuity and potentially reducing iodine contrast dose [66]. Similar to single-energy low-kVp CT, DECT is associated with increased image noise and therefore relies heavily on advanced reconstruction techniques, including iterative and deep learning-based algorithms, to maintain diagnostic image quality [67, 68]. Several studies have demonstrated that DECT can achieve radiation dose levels comparable to or lower than conventional single-energy CT while providing additional capabilities such as material decomposition and virtual monoenergetic imaging [69, 70].

### Research gaps

Despite encouraging evidence supporting the use of low-kVp techniques for radiation dose reduction in CECT abdominopelvic, several critical research gaps remain. Current studies show substantial heterogeneity in acquisition parameters, reconstruction methods, and reporting of dose metrics, which limits comparability and reproducibility. To accurately evaluate and compare the effectiveness of dose reduction, it is essential to clearly define the specific dose metrics being used and move toward standardized reporting. Most evidence originates from single-center studies, and there is a lack of large, prospective, multicenter validation in routine clinical practice. Many important clinical indications, including urologic and gynaecological malignancies, inflammatory bowel disease, trauma, and detection of small or low-contrast lesions, remain underrepresented. Furthermore, most studies emphasize image quality metrics rather than diagnostic accuracy outcomes such as sensitivity, specificity, and lesion detectability. Importantly, there is a lack of task-based assessment in current research, with limited evaluation of clinically relevant detection tasks. Future studies should incorporate task-based evaluation frameworks to better assess diagnostic performance and lesion detectability in low-kVp CT imaging. Patient size stratification is inconsistently applied, relying mainly on BMI, while alternative body habitus parameters are rarely explored, and evidence in overweight or obese populations is limited. The clinical impact of automatic tube voltage selection systems and their interaction with reconstruction techniques and contrast dosing remains insufficiently defined. Although deep learning–based reconstruction shows promise, validation is currently limited to small, vendor-specific cohorts. Finally, no studies have evaluated the feasibility, cost-effectiveness, or protocol optimization frameworks that integrate patient characteristics, clinical indications, kVp selection, reconstruction methods, and contrast doses. These gaps highlight the need for standardized reporting, multicenter clinical validation, and outcome-based research to support the safe and widespread implementation of low-kVp protocols in abdominopelvic CT.

### Limitation

A key limitation of this scoping review lies in the heterogeneity of the included studies. There was significant variability in CT scanner models, imaging protocols, sample sizes, and reconstruction techniques, which makes direct comparisons between studies challenging and limits the ability to draw consistent conclusions. Additionally, the lack of standardization in reporting radiation dose metrics such as CTDI_vol_, DLP, and effective dose, as well as the use of varying methods for assessing image quality, further complicates the synthesis and generalizability of the findings. Moreover, most included studies were conducted in controlled settings or single institutions, which may limit generalizability to routine clinical practice across diverse healthcare environments. Finally, this review focused solely on CECT in adults, thereby excluding findings that may apply to non-contrast protocols or paediatric populations.

#### Recommendations

To enhance the comparability and clinical utility of future research, there is a strong need for standardization in reporting protocols. Studies should adopt uniform methods for documenting radiation dose metrics, image quality parameters, and reconstruction settings to enable more consistent interpretation and reproducibility of results. Additionally, comprehensive image quality evaluation should prioritize both objective measures, such as SNR, CNR, and noise, as well as subjective assessments through radiologist grading to ensure diagnostic reliability. Incorporating patient-specific indicators such as BMI, weight, or abdominal diameter into manual or automated kVp selection strategies is crucial for achieving an optimal balance between radiation dose reduction and image quality preservation. Additionally, the development and integration of clinical decision support tools that automate kVp selection based on individual patient characteristics and clinical indications may support protocol consistency, enhance workflow efficiency, and promote the routine adoption of personalized low-dose CT imaging protocols.

## Conclusion

This scoping review mapped the existing literature on the application of low tube voltage techniques for radiation dose optimization in adult CECT abdominopelvic CT. The available evidence shows a growing trend toward the use of low-kVp protocols in combination with iterative and deep learning–based reconstruction methods, with most studies reporting notable reductions in radiation dose and improvements in conventional image quality metrics. However, the evidence base remains heterogeneous with respect to study design, scanner technology, acquisition parameters, reconstruction algorithms, and outcome measures. As a scoping review, this work does not establish the definitive effectiveness of any specific technique, but rather highlights prevailing practices, methodological variability, and areas where evidence is limited. The findings emphasize the need for standardized reporting, greater use of task-based diagnostic accuracy outcomes, and large prospective multicenter studies to define the clinical boundaries and optimal application of low-kVp strategies in abdominopelvic CT.

## Data Availability

Not available.
